# The Association Between Physical Activity and Obesity: A Systematic Review

**DOI:** 10.7759/cureus.99068

**Published:** 2025-12-12

**Authors:** Murwan Mageit, Mohammed Yossef Alhabib, Mohammed Alshamrani, Adel AlShahrani, Rakan AlShaalan, Huda AlAnazi, Wejdan Alharbi, Arwa S AlObaid, Saqer Abdullah AlHarthi

**Affiliations:** 1 Department of Neurology, University of Khartoum, Khartoum, SDN; 2 Department of Family Medicine, Prince Sultan Military Medical City, Riyadh, SAU; 3 Department of Anesthesiology, Prince Sultan Military Medical City, Riyadh, SAU; 4 Department of Family Medicine, Buraydah General Hospital, Al-Qassim, SAU; 5 Department of Family Medicine, Alrass General Hospital, Al-Qassim, SAU; 6 Department of Family Medicine, Family Medicine Academy, Al-Qassim, SAU; 7 Department of Physical Medicine and Rehabilitation, Prince Sultan Military Medical City, Riyadh, SAU

**Keywords:** correlation, metabolic diseases, obesity, overweight, physical activity

## Abstract

Obesity and overweight represent major public health challenges due to their widespread prevalence and associated adverse outcomes. Substantial evidence highlights the protective role of physical activity in mitigating these conditions and related health risks. This systematic review seeks to examine the relationship between physical activity and obesity. This systematic review was conducted in accordance with the Preferred Reporting Items for Systematic Reviews and Meta-Analyses (PRISMA) guidelines. A comprehensive search was performed in PubMed, Scopus, and Web of Science using the terms “adult,” “overweight,” “obesity,” “physical activity,” and “exercise.” The review analyzed the relationship between physical activity and obesity-related indicators, including BMI, waist circumference, and body fat percentage, across human populations of varying age groups. The findings of this review support an inverse association between physical activity levels and both the prevalence and incidence of obesity across diverse age groups and populations. Regular engagement in physical activity is associated with lower obesity risk, improved body composition, and favorable metabolic outcomes, whereas sedentary behavior emerged as a key modifiable risk factor. To mitigate the global burden of obesity, public health strategies should prioritize policies and interventions that encourage sustained physical activity from early life stages.

## Introduction and background

Obesity and overweight refer to the abnormal and excessive accumulation of fat, which can have negative effects on health. The global incidence of obesity is increasing; by 2030, 3.3 billion adults, or 57.8 percent of the population, will be classified as overweight [1]. Obesity is influenced by a variety of factors, such as genetic predisposition, lack of physical activity, dietary choices, and behavioral factors [2]. There is a growing epidemic of obesity around the world. Being overweight increases the likelihood of developing numerous significant chronic diseases, such as heart disease, dyslipidemia, type 2 diabetes, high blood pressure, cancer, and osteoarthritis. Overweight or obesity is the fifth most common cause of mortality, resulting in the deaths of at least 2-8 million adults annually [3]. Healthy nutrition provides substantial health benefits. A nutritious diet is composed of a diverse array of natural, fresh foods, such as fruits and vegetables, and provides essential minerals and vitamins. Moreover, consistent physical activity and healthy dietary practices promote both physical and emotional well-being [4]. Adolescents are particularly vulnerable to eating disorders. In the past, obesity-related eating disorders were assessed utilizing the Three-Factor Eating Questionnaire (TFEQ). Subsequently, a revised questionnaire was developed, which comprises three components: “cognitive restraint,” “uncontrolled eating,” and “emotional eating” [5].

The prevalence of two lifestyle behaviors is a strong predictor of metabolic health, obesity, chronic illnesses including type 2 diabetes and cardiovascular diseases (CVD), and overall mortality: inactivity and a sedentary lifestyle [6]. The term “physical activity” refers to bodily movement facilitated by skeletal muscles that results in energy expenditure above the resting level. Movements that are light, moderate, or vigorous in intensity are considered physical activity. To be considered physical activity, a metabolic equivalent (MET) value of at least 1.6 units is required. In turn, sedentary behavior, also known as “sitting,” is characterized by minimal movement and is associated with an energy expenditure of 1.5 METs or less [7]. Sedentary behavior has the potential to influence health separately through various mechanisms, including dysregulation of lipoprotein lipase activity and inflammation, or simply by displacing moderate-to-vigorous physical activity (MVPA) [8]. Excess weight gain and obesity can be influenced by modifiable risk factors, one of which is an inactive lifestyle. Contrarily, the fact that regular physical activity has major positive effects on health is well known [9]. Overweight and obesity are among the many health problems that may benefit from increased physical activity, according to a growing body of research [10]. Higher life expectancy and a life free of diabetes are two outcomes associated with regular physical exercise. Being inactive raises the risk of obesity since it negatively affects body weight status [11]. The correlation between inactivity and weight gain has been the subject of a great deal of research, including both observational studies and randomized controlled trials (RCTs). Variations in study design, demographics, physical activity intensity and type, and obesity assessment methodologies have led to contradictory results. The objective of this systematic review was to evaluate and synthesize the current research on the relationship between physical activity and obesity in order to ascertain the extent to which physical exercise improves obesity-related outcomes across various demographics.

## Review

Methods

The Preferred Reporting Items for Systematic Reviews and Meta-Analysis (PRISMA) procedure was adhered to in this systematic review [12].

Data sources and search strategy

Electronic databases such as PubMed, Scopus, and Web of Science were searched using keywords including “physical activity,” “exercise,” “adult,” “overweight,” and “obesity.” The electronic searches were restricted to studies published in English and involving human subjects. Additionally, we conducted a manual evaluation of the reference lists from the included clinical trials and prior reviews to identify potentially relevant studies.

Eligibility criteria

Inclusion Criteria

We included peer-reviewed articles of different study designs conducted in overweight or obese individuals of all ages to assess how physical activity correlated with obesity-related metrics such as BMI, waist circumference, and body fat percentage. We considered studies that measured physical activity clearly and reported direct outcomes associated with overweight or obesity.

Exclusion criteria

We excluded studies for the following reasons: (1) case reports, case series, editorials, commentaries, conference abstracts, reviews, meta-analyses, or non-peer-reviewed articles; (2) studies that did not clearly measure physical activity or those in which physical activity was combined with other interventions (e.g., pharmacological treatment) without the ability to isolate its independent effect; and (3) non-English articles.

Data extraction

Data extraction was conducted manually by two independent authors using a structured form. The collected data included the first author’s name, study location, study design, publication year, and sample size, in addition to participant demographic data such as age and sex. Additionally, the outcomes of interest and the key findings of included studies were extracted.

Quality assessment

Two authors independently evaluated the risk of bias of the included studies using the Newcastle-Ottawa Scale (NOS) to assess the quality of observational cohort studies (total score: 9 points). Total scores of 7-9 points were rated as high quality; 5-6 points were considered fair or moderate quality; and scores of 4 points or less were rated as poor quality [13]. Regarding cross-sectional studies, we used the AXIS critical appraisal tool to assess study quality [14].

Results

Search Results

**Figure 1:**
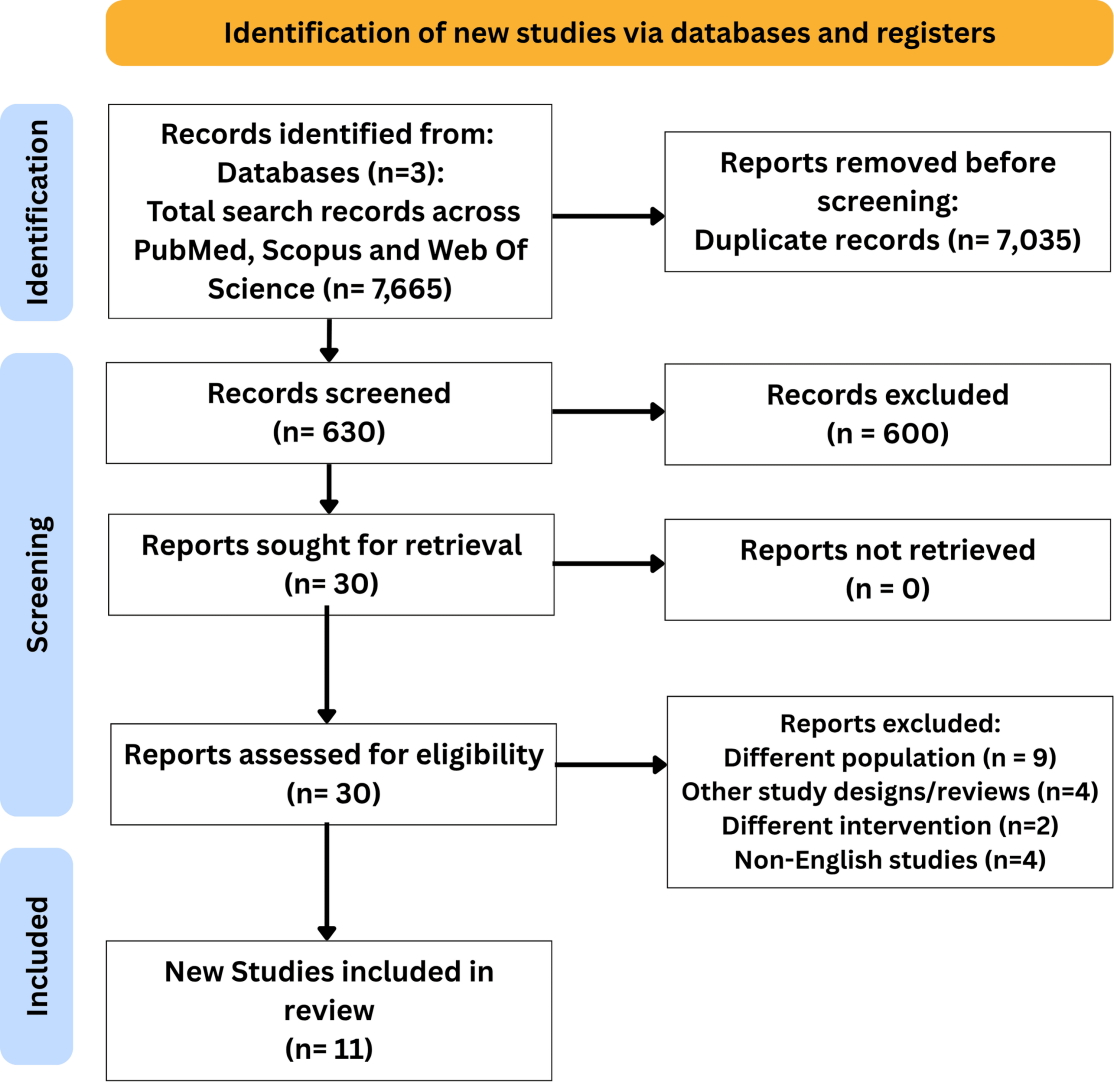
PRISMA flow diagram of included studies. PRISMA: Preferred Reporting Items for Systematic Reviews and Meta-Analyses.

During the initial search, 7665 records were found. After excluding duplicates, a total of 630 records underwent title and abstract screening. Of the 630 records, 600 articles were excluded according to predefined eligibility criteria. The remaining 30 full-text articles were assessed for eligibility. Finally, eleven articles were eligible for qualitative synthesis (Figure 1).

Study Characteristics

Our comprehensive systematic review included 11 studies [13-23] encompassing diverse populations, study designs, and geographic regions (Table 1). Specifically, the studies represented a broad geographic distribution, including the United Kingdom, Australia, the USA, the Netherlands, Egypt, Poland, Jordan, and a global multi-country cohort. Furthermore, total sample sizes ranged from small observational samples (n = 71 participants) to large-scale cross-sectional studies involving over 280,000 adolescents worldwide, and covered children, adolescents, adults, and older adults. In terms of methodology, the review incorporated three cohort studies [13,14,17], one longitudinal study [15], and seven cross-sectional studies [16,18,23], thereby providing a comprehensive overview of the relationship between physical activity and obesity across different life stages. Collectively, the evidence indicated that higher levels of physical activity were consistently associated with lower obesity risk and improved body composition. Conversely, physical inactivity and sedentary behavior were linked to a greater likelihood of overweight and metabolic disorders, irrespective of age or demographic factors (Table 2).

**Table 1:** Baseline characteristics of the studies included in this systematic review.

Study ID	Year	Country	Study design	Sample size	Age (years)	Sex
Bell JA et al. [15]	2014	UK	Cohort study	3,670 participants	55.5 ± 6.0 years	73% males
Montgomerie AM et al. [16]	2014	Australia	Biomedical cohort study	1,521 participants	44.6 ± 16.22 years	50.6% males
Pavey TG et al. [17]	2016	Australia	Longitudinal study	2,735 women	24.6 (20.6-28.5) years	100% women
Hong I et al. [18]	2016	USA	Cross-sectional	1,640 children	3-15 years	49.8% females
Koolhaas CM et al. [19]	2017	The Netherlands	Prospective cohort study	5,344 participants	68.5 ± 7.9 years	60.1% women
Abd El-Shaheed A et al. [20]	2020	Egypt	Cross-sectional	89 adolescents	10-18 years	66.3% females
Mahumud RA et al. [21]	2021	Global study	Cross-sectional	282,213 adolescents	11-17 years	51.52% females
Zaki M and Youness ER [22]	2022	Egypt	Cross-sectional	600 subjects	13-17 years	58% females
Liu Y et al. [23]	2024	Poland	Cross-sectional	71 women	68.8 ± 4.3 years	100% women
Batiha AM et al. [24]	2024	Jordan	Descriptive cross-sectional study	1,554 participants	14-18 years	54% males
Nowara AS et al. [25]	2025	Egypt	Cross-sectional	368 medical students	22.19 ± 1.66 years	64.4% males

**Table 2:** Main findings of the included studies.

Study ID	Outcome of interest	Main findings
Bell JA et al. [15]	Incident obesity	The analysis revealed a significant association between physical activity and incident obesity. Specifically, individuals who maintained high levels of physical activity combined with low amounts of leisure-time sitting exhibited the lowest risk of developing obesity after five years (OR = 0.26; 95% CI: 0.11-0.64). However, this protective effect appeared to decline over a ten-year follow-up period.
Montgomerie AM et al. [16]	Incident obesity	Incident obesity was found to be associated with physical inactivity (RR = 1.48; 95% CI: 1.14-1.90 (SAMSS) and RR = 1.41; 95% CI: 1.03-1.93 (NWAHS)). However, this relationship weakened after adjusting for potential covariates. In contrast, physically inactive individuals had approximately a 41-48% higher risk of obesity (RRs = 1.41 and 1.48, respectively) compared with their active counterparts.
Pavey TG et al. [17]	Change in BMI category	The findings indicated that maintaining extremely high physical activity levels reduced the risk of becoming overweight or obese during young adulthood. This supports sustained physical activity as a preventive strategy against weight gain in early life stages.
Hong I et al. [18]	Childhood overweight and obesity	The odds ratio (OR) for obesity was 0.93 (95% CI: 0.87-0.98) when comparing the two physically active groups, indicating a protective effect of activity. However, although both overweight and normal-weight children engaged in physical activity, the overweight group experienced more pronounced functional limitations, which may have influenced the extent of their participation.
Koolhaas CM et al. [19]	BMI categories, physical activity levels, and incidence of CVD events	Overweight and obese individuals who exercised infrequently were at higher risk of developing cardiovascular disease (CVD) compared with normal-weight individuals who engaged in regular physical activity. This suggests that insufficient exercise, rather than weight status alone, contributes substantially to elevated CVD risk.
Abd El-Shaheed A et al. [20]	Pattern and intensity of physical activity among obese and non-obese adolescents	Physical activity habits and patterns differed between obese and non-obese Egyptian adolescents. Obese adolescents reported higher levels of activity, particularly aerobic exercise, which may reflect compensatory efforts to manage weight or meet health recommendations.
Mahumud RA et al. [21]	Incidence of overweight/obesity; association of lifestyle risk factors (nutrition, physical activity, sedentary behavior) with overweight/obesity	A significant association was observed between unhealthy eating habits, physical inactivity, and the likelihood of being overweight or obese (RRR = 1.11; 95% CI: 1.06-1.17 and RRR = 1.20; 95% CI: 1.12-1.28, respectively). These findings suggest that poor dietary patterns combined with insufficient physical activity and prolonged sedentary behavior contribute to overweight and obesity among adolescents.
Zaki M and Youness ER [22]	Metabolic syndrome (MS), dyslipidemia, and obesity in relation to physical activity	Physical inactivity was strongly associated with a higher risk of metabolic syndrome (MS), dyslipidemia, and obesity among adolescents, underscoring the role of active lifestyles in preventing metabolic and weight-related disorders.
Liu Y et al. [23]	Obesity indicators (visceral fat, BMI, waist circumference, body fat %)	Postmenopausal women who engaged in at least 150 minutes of moderate-to-vigorous physical activity (MVPA) per week demonstrated lower obesity indices. Higher activity was linked to improved body composition, more favorable energy metabolism hormone profiles, and a reduced risk of metabolic syndrome. Increasing physical activity while reducing sedentary time may help prevent metabolic syndrome and cardiovascular disease (CVD) in this population.
Batiha AM et al. [24]	Overweight and obesity in relation to exercise	The prevalence of overweight was higher (12%) among individuals who did not participate in any regular physical activity program. However, although no statistically significant association was observed between the overall amount of physical activity and weight status, the prevalence of overweight and obesity remained elevated across levels of sport participation.
Nowara AS et al. [25]	Incidence of overweight and obesity	Medical students were found to be at considerable risk of overweight and obesity. Overweight and obese students reported significantly lower levels of physical activity compared with their non-overweight peers, suggesting that insufficient activity may contribute to excess weight in this population.

Quality Assessment

The risk of bias assessment of four cohort and observational studies using the NOS tool showed overall good quality, with total scores ranging between 7 and 9 points (Table 3). Regarding the seven cross-sectional studies evaluated using the AXIS tool, all included studies showed a low risk of bias with acceptable quality, as shown in Table 4.

**Table 3:** Quality assessment of cohort studies using the Newcastle-Ottawa Scale.

Study ID	Selection				Comparability	Outcome			Total score
	Representativeness of the exposed cohort	Selection of the non-exposed cohort	Ascertainment of exposure	Demonstration that outcome of interest was not present at start of study	Comparability of cohorts on the basis of the design or analysis	Assessment of outcome	Was follow-up long enough for outcomes to occur	Adequacy of follow-up of cohorts	Total score
Bell JA et al. [15]	*	*	*	*	*	*	-	*	7
Montgomerie AM et al. [16]	*	*	*	*	**	*	*	*	9
Pavey TG et al. [17]	*	*	*	*	*	*	*	*	8
Koolhaas CM et al. [19]	*	*	*	*	*	*	*	*	8

**Table 4:** Quality appraisal of cross-sectional studies using the AXIS tool. AXIS: Appraisal tool for Cross-Sectional Studies

Study ID	Clear aim	Appropriate design	Adequate sample size	Clear population	Representative sample	Appropriate sampling	Addressed non-response bias	Direct data collection	Valid exposure measures	Valid outcome measure	Appropriate methods	Described statistics	Addressed risk and confounding factors	Consistent and discussed results	Certainty estimation	Addressed limitations
Hong I et al. [18]	Yes	Yes	Yes	Yes	Yes	Yes	Partially	Yes	Yes	Yes	Yes	Yes	Yes	Yes	Yes	Yes
Abd El-Shaheed A et al. [20]	Yes	Yes	Yes	Yes	Yes	Yes	No	Yes	Yes	Yes	Yes	Yes	Yes	Yes	Partially	Yes
Mahumud RA et al. [21]	Yes	Yes	Yes	Yes	Yes	Yes	No	Yes	Yes	Yes	Yes	Yes	Yes	Yes	Yes	Yes
Zaki M and Youness ER [22]	Yes	Yes	Yes	Yes	Yes	Yes	Partially	Yes	Yes	Yes	Yes	Yes	Yes	Yes	No	Partially
Liu Y et al. [23]	Yes	Yes	Yes	Yes	Yes	Yes	Yes	Yes	Yes	Yes	Yes	Yes	Yes	Yes	Yes	Yes
Batiha AM et al. [24]	Yes	Yes	Yes	Yes	Yes	Yes	Partially	Yes	Yes	Yes	Yes	Yes	Yes	Yes	Partially	Yes
Nowara AS et al. [25]	Yes	Yes	No	Yes	Yes	Yes	Yes	Yes	Yes	Yes	Yes	Yes	Yes	Yes	Partially	Yes

Discussion

The association between inactivity and excess body fat was investigated in this meta-analysis. Physical inactivity and sedentary behavior were associated with an increased risk of overweight, obesity, and related metabolic diseases across all age groups and demographic subgroups, whereas increased physical activity was associated with improved body composition and a decreased risk of obesity. The study by Bell JA et al. [15] examined the cumulative effect of prolonged sitting and moderate-to-vigorous physical activity on the risk of obesity and metabolic risk factors over time. They found that leisure-time sitting was associated with overweight, whereas being physically active was associated with a lower risk of obesity. The odds of developing obesity were lowest after five years for people who were very active and did not sit for long periods of time (OR = 0.26; 95% CI 0.11, 0.64), and they were even lower after ten years. After five years, there was a decreased likelihood of metabolic risk factor clustering for those who fell somewhere in the middle of the spectrum regarding their levels of physical activity and leisure-time sitting (OR 0.53; 95% CI 0.36, 0.78), and the odds remained similar after ten years. According to their research, if we want to significantly lower the risk of obesity, we may need to do more exercise and spend less time sitting [15].

In a study conducted by Boone JE et al. [26] on young adults, it was found that those who were more active and had lower screen-based sitting time had a lower chance of becoming overweight or obese after five years. However, this link was only evident in females [26]. It is not immediately clear what processes are at work in this connection. Theoretically, less sitting during leisure time may augment the beneficial effects of greater physical activity, either because of separate physiological mechanisms or because it reflects more participation in low-intensity activities such as standing [27]. An elevation in sedentary behavior correlated with the aggregation of metabolic risk factors in several cross-sectional investigations [28,29], regardless of physical activity levels; however, other research show that the strength of this link depends on activity levels [30,31]. Multiple prospective studies have shown that, regardless of whether participants engaged in moderate-to-vigorous physical activity, increased sitting time was linked with worsening insulin profiles [32] and the clustering of metabolic risk variables [33]. In contrast, additional research has shown that, over the course of several years of follow-up, more TV viewing time [34] and decreased physical activity independently predicted worse metabolic profiles.

The correlation between obesity and CVD risk in middle-aged and older adults was investigated in a prospective study by Koolhaas CM et al. [19] according to their levels of physical activity. People who were overweight or obese and did not exercise much were more likely to develop CVD than people of a healthy weight who exercised frequently. According to their research, CVD prevention benefits from exercise may exceed those of a healthy BMI in middle-aged and older adults. This shows that people of all weights should be active, and that being sedentary is dangerous even for those with a normal BMI.

In addition, a trial involving 18,892 men and women from Finland ranging from 25 to 74 years old found that being physically inactive is related to an increased risk of CVD on its own, but being overweight raises the risk by influencing other risk factors [35]. In accordance with the findings of the Women’s Health Study, higher levels of physical activity significantly reduce the risk of coronary heart disease associated with a higher body mass index [36]. However, the risk was not entirely eliminated, emphasizing the importance of maintaining a lean physique and participating in consistent physical activity [36]. Likewise, data from the Nurse’s Health Study involving 88,393 women aged 34 to 59 years indicated that moderate physical activity mitigated, but did not eradicate, the detrimental impact of obesity on the risk of coronary heart disease. They also demonstrated that maintaining a lean physique did not mitigate the heightened risk linked to physical inactivity [37].

Extensive research has been conducted on the negative impact of overweight and obesity on the risk of CVD. Increased inflammation, atherosclerosis, endothelial dysfunction, and coagulation problems are some of the ways in which adipose tissue affects cardiac function through the release of interleukins, free fatty acids, and cytokines [38]. The CVD risk benefits linked to physical activity are thought to be caused by a decrease in myocardial oxygen demand, stabilization of vulnerable plaques (reducing the risk of plaque rupture), and improved endothelial function. It may be deduced from this that engaging in physical activity has the ability to directly mitigate and counteract the detrimental impact of prothrombotic substances generated by adipose tissue [36].

## Conclusions

In conclusion, this systematic review revealed that increased physical activity levels were negatively correlated with the prevalence and incidence of obesity among various populations. Consistent physical exercise lowered obesity risk, improved body composition, and produced beneficial metabolic outcomes, whereas a sedentary lifestyle and lack of physical activity remained substantial modifiable risk factors for obesity and its related diseases. Future research should emphasize longitudinal studies utilizing objective assessments of physical activity and adiposity, while also accounting for contextual and socio-environmental factors that may affect activity patterns. The creation and execution of evidence-based initiatives that encourage continuous physical activity from early childhood to adulthood are crucial to alleviating the worldwide obesity burden.
